# Prediction of Premature Termination Codon Suppressing Compounds for Treatment of Duchenne Muscular Dystrophy Using Machine Learning

**DOI:** 10.3390/molecules25173886

**Published:** 2020-08-26

**Authors:** Kate Wang, Eden L. Romm, Valentina L. Kouznetsova, Igor F. Tsigelny

**Affiliations:** 1 MAP program, University of California San Diego (UCSD), La Jolla, CA 92093, USA; katewang2003@gmail.com; 2 Curematch Inc., 6440 Lusk Blvd, Suite D206, San Diego, CA 92121, USA; eromm@curematch.com; 3 San Diego Supercomputer Center, University of California San Diego (UCSD), La Jolla, CA 92093, USA; vkouznesova@ucsd.edu; 4 Dept. of Neurosciences, University of California San Diego (UCSD), La Jolla, CA 92093, USA

**Keywords:** Duchenne muscular dystrophy, stop codon, machine learning, deep learning, pharmacophore, PTC-suppressing compounds

## Abstract

A significant percentage of Duchenne muscular dystrophy (DMD) cases are caused by premature termination codon (PTC) mutations in the dystrophin gene, leading to the production of a truncated, non-functional dystrophin polypeptide. PTC-suppressing compounds (PTCSC) have been developed in order to restore protein translation by allowing the incorporation of an amino acid in place of a stop codon. However, limitations exist in terms of efficacy and toxicity. To identify new compounds that have PTC-suppressing ability, we selected and clustered existing PTCSC, allowing for the construction of a common pharmacophore model. Machine learning (ML) and deep learning (DL) models were developed for prediction of new PTCSC based on known compounds. We conducted a search of the NCI compounds database using the pharmacophore-based model and a search of the DrugBank database using pharmacophore-based, ML and DL models. Sixteen drug compounds were selected as a consensus of pharmacophore-based, ML, and DL searches. Our results suggest notable correspondence of the pharmacophore-based, ML, and DL models in prediction of new PTC-suppressing compounds.

## 1. Introduction

Duchenne muscular dystrophy (DMD) is an X-linked neuromuscular disorder characterized by progressive muscular degeneration. DMD affects about 1 in 3500 male births worldwide [1]. Currently, dystrophin is considered one of the most important genes involved in DMD. This protein is a component of the dystrophin-associated protein complex (DAPC) [1,2]. Mutations in the dystrophin gene on the X chromosome cause DMD; they damage the DAPC, causing creatine kinase to pass through muscle cell membranes into the blood, in turn, leading to elevated serum creatine kinase levels [2].

About 5–15% of DMD cases are caused by nonsense mutations, resulting in premature termination codons (PTCs) in the place of a sense codon. This leads to the production of a truncated dystrophin protein [2,3]. Consequently, several therapeutic approaches for treating DMD have focused on suppressing PTCs. One therapeutic approach in particular—the use of PTC-suppressing compounds (PTCSCs)—relies on the fact that translation termination is not 100% efficient [3]. It depends on competition between the human eFR1 release factor, which recognizes the stop codon, and near-cognate aminoacyl-tRNAs, which carry anticodons with an altered third nucleotide base [3]. Binding of near-cognate aminoacyl-tRNAs to the stop codon causes an amino acid to be incorporated, resulting in the continuous production of protein, as there is no longer a stop codon to be recognized [3]. Thus, PTCSCs suppress PTCs by inducing translational readthrough, a mechanism that leads to the production of full-length protein by stimulating the binding of near-cognate tRNAs, to the stop codon [3].

PTCSCs are hypothesized to bind to the 30S ribosomal subunit and interact with the 16S rRNA and S12 ribosomal protein [4]. The 30S ribosomal subunit plays an important role in discriminating against near cognate aminoacyl tRNAs, ensuring translation accuracy. Binding of PTC-suppressing compounds to the 30S ribosomal subunit inhibits this process, resulting in codon misreading and altered translation of mRNA [4]. Our hypothesis is that some ribosomal proteins have a high degree of homological correspondence to the analogous human ribosomal proteins, explaining the efficacy of these antibiotics for suppression of PTCs in humans.

One class of PTCSCs is aminoglycoside antibiotics. Aminoglycosides, such as gentamicin and paromomycin, have been found to stimulate readthrough and suppress PTCs. However, the use of these drugs is limited, as they may induce ototoxicity and nephrotoxicity, manifesting in side effects such as hearing loss and diminished renal function [5]. Their efficacy is also influenced by other factors, such as stop codon context and sequence specificity. Previous studies have found that readthrough levels induced by aminoglycosides have the order UGA > UAG >> UAA, and a +4 cytosine immediately following the PTC produced the most readthrough, due to the prevention of eRF1 recognition of the PTC [5]. Aminoglycoside derivatives meant to reduce toxicity and improve therapeutic potential were developed by modifying the chemical structures of aminoglycosides [6]. As examples of such drug-candidates, we can list NB54 and NB30, which are paromomycin derivatives, along with NB74 and NB84, which are geneticin (G418) derivatives [6,7].

Non-aminoglycosidic alternatives have also been identified through screens of compounds [8]. One such drug-candidate is Ataluren, or PTC124, a PTC-suppressing oxadiazole compound of lower toxicity. Ataluren has been proposed to stimulate readthrough by binding to the h44 decoding center of the 16S rRNA in the 30S ribosomal subunit [9]. While this drug has been found to have significantly lower toxicity, conflicting results have been found in regard to its efficacy [10,11]. Another PTC-suppressing compound, negamycin, exhibited lower toxicity than aminoglycosides [11]. Negamycin follows a similar mechanism of action to aminoglycosides, binding to the ribosomal A site. [11]. Furthermore, macrolides, antibiotics used to treat bacterial infections, have been tested as readthrough-inducing compounds [12]. This family of compounds includes tylosin, josamycin, spiramycin, and azithromycin [12,13]. Macrolides were able to induce readthrough of nonsense mutations, indicating that they could be potential PTC-suppressing agents [14]. Additional non-aminoglycosides that have been investigated include clitocine, escin, RTC #13, RTC #14, GJ071, GJ072, and amlexanox [15,16,17,18,19,20,21]. Clitocine is an adenosine nucleotide analog [15]. In contrast to aminoglycosides, clitocine is incorporated in replacement of the adenine in a stop codon, inducing readthrough [15]. However, this compound is also affected by stop codon context, with the order of clitocine-induced readthrough levels for various PTCs being UAA >> UGA > UAG [15]. Escin is an herbal anti-inflammatory drug that was able to induce read-through in a cystic fibrosis patient [16]. This compound may be a potential PTC-suppressing compound used to treat DMD as well [16]. High-throughput screening identified potential readthrough-inducing compounds RTC #13 and RTC #14 [17,18]. Studies have shown that RTC #13 was able to partially restore dystrophin levels in the muscles of mdx mice [18]. More recently, GJ071 and GJ072 showed a similar read-through-inducing efficiency as PTC124, RTC #13, and RTC #14, as well as lower toxicity [19].

Computational approaches for identification of PTCSCs help to expedite the reduction of large molecular databases to the sets of molecules which are most probable to exhibit an optimized blend of features to be tested in vitro; inhibitors which are efficacious and low in toxicity. Only one paper describing the deployment or development of such screens for DMD was found [22], despite the potential development of such screens and their widespread use in the search for drugs to treat other diseases. Yusuke et al. developed an in-silico tool that can design nucleotide analogs which recognize, bind, and block transcription or splice sites of pre-mRNAs, morpholino sequences, for exon skipping [22]. Remarkably, they report that most of their computationally derived morpholinos are more efficient at promoting Exon 51 skipping in vitro than Eteplirsen [22], the current FDA approved treatment of this type for this disease.

Computational drug design employs different methods. Pharmacophore-based methods have proven to be some of the most effective for computational drug design approaches. Researchers create a set of functional centers based on either ligand or receptor and use it for selection from conformational databases of compounds. Another popular, modern method uses quantitative descriptions of already existing agonists or antagonists of the specific proteins, called quantitative structure-activity relationship (QSAR) descriptors, to create a machine learning (ML) model that would be used for the elucidation of new compounds that share this activity on the target protein.

Here, we point out some successful stories of using pharmacophore-based and ML-based searches for new drug design.

In one study, an inhibitor of the promising drug target acid sphingomyelinase (ASM) was selected using a pharmacophore model created on the basis of known ASM inhibitors including α-mangostin [23]. A database search using this pharmacophore model revealed 23 potential inhibitors, 10 of which were found to be effective in experimental studies.

A machine learning system for elucidation of potential drug candidates for the 5-HT_2B_ receptor (5-hydroxytryptamine receptor 2B) inhibition was developed using known inhibitors with Ki < 500 nM and inactive molecules with Ki > 1000 nM. The author of this study used a NSFP fingerprint-based ML method and virtual docking of the obtained compounds to the binding sites. Nine potential inhibitors were selected, five of which showed binding to the 5-HT_2B_ receptor and one with Ki = 0.3 mM [24]. In the same study, Bruns used various machine learning algorithms, including self organizing maps (SOM), convolution neural networks (CNN), and recurring neural networks (RNN), for development of novel inhibitors of the CXC chemokine receptor 4 (CXCR4) based on known inhibitors and non-inhibitor compounds. With the most effective SOM ML system, he elucidated a new inhibitor with an EC_50_ (half maximal effective concentration) < 10μM.

In another study, Li and colleagues [25] conducted classical ML-based elucidation of new inhibitors of topoisomerase I. They prepared an input set containing 481 inhibitors and 480 non-inhibitor compounds. They used 189 molecular descriptors, k-nearest neighbors (KNN), radio frequency (RF), and support vector machines (SVM) ML methods to develop their models and conducted further virtual screening of the Maybridge database. Following selection, molecular docking was conducted with AutoDock Vina software [26]. The authors elucidated several compounds with docking energies better than −10 kcal/mol and similar scaffolds to known topoisomerase I inhibitors.

In our recent study [27], we attempted to compare the results of pharmacophore-based and ML-based drug design, and confirmed that results of pharmacophore-based and deep learning (DL)-based drug selection were similar in a significant part of the predicted drug candidates.

In the current study, we also attempted to concentrate on the pharmacophore-based, DL, and ML-based techniques for predictions of the same compounds, which we can consider the more robust predictions for further testing.

Here, we deployed pharmacophore-modeling along with ML and DL based approaches to allow for identification of novel drug candidates with PTC-suppressing ability for the treatment of DMD. A pharmacophore model was developed based on common pharmacophore features of existing PTC-suppressing compounds and used to screen for compounds with structural correspondence to these pharmacophore features. To validate pharmacophore-based results, ML and DL models were developed for prediction of PTC-suppressing compounds using QSAR descriptors. This study develops an ensemble of models to predict new compounds with PTC-suppressing ability and lays the foundation for future investigation of the identified PTC-suppressing drug candidates for treatment of DMD.

## 2. Results

### 2.1. Similarity Clustering

Similarity clustering with the parameter GpiDAPH3 45% exposed two clusters of compounds populated by seven compounds or more. Cluster A included eleven compounds: gentamicin, tobramycin, neomycin, streptomycin, geneticin, amikacin, paromomycin, apramycin, hygromycin B, kanamycin A, and plazomicin. These compounds are aminoglycosides, characterized by amino sugars connected to a dibasic aminocyclitol. Cluster B contained seven compounds: josamycin, spiramycin, erythromycin, azithromycin, GJ072, escin, and amlexanox.

### 2.2. Flexible Alignment

**Figure 1:**
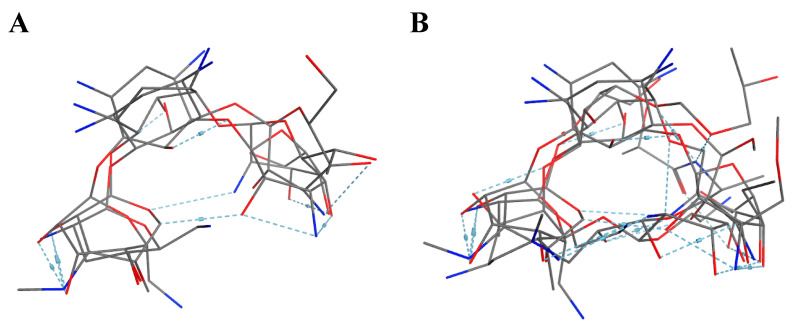
Flexible alignment with carbon atoms in gray, oxygen atoms in red, and nitrogen atoms in blue. (**A**) Compounds **1**–**4** in Cluster A, (**B**) compounds **1**–**7** in Cluster B.

10 flexible alignment structures for Cluster A and 19 flexible alignment structures from Cluster B were produced. The flexible alignments appearing the most two-dimensional and showing the greatest overlap of the compounds’ chemical structures were selected for each cluster. The structures, as shown in the figures below, contain three rings (Figure 1).

### 2.3. Development of a Pharmacophore Model

**Figure 2:**
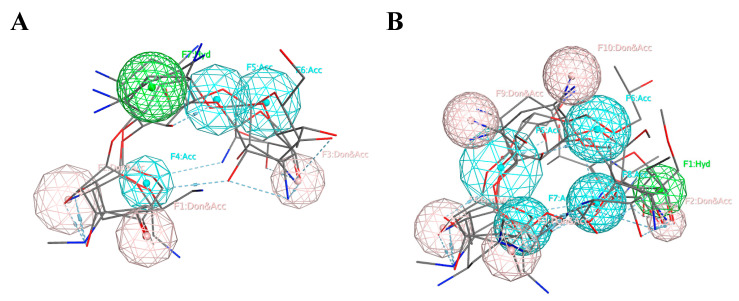
The pharmacophore models created using the pharmacophore centers donor/acceptor (Don/Acc), acceptor (Acc), and hydrophobic (Hyd), represented by pink, blue, and green, respectively. (**A**) Cluster A, (**B**) Cluster B.

Based on the alignment of Cluster A, we created a seven-feature pharmacophore model. The pharmacophore model is described by three donors/acceptors, three acceptors, and one hydrophobic region. Based on the alignment of Cluster B, we developed a five-feature pharmacophore model. The five features consisted of three donors/acceptors, one hydrophobic region, and one acceptor. Three acceptors (Acc) were added to regions containing a high density of oxygen atoms, and two donors/acceptors (Don/Acc) were added to regions containing a high density of oxygen and nitrogen atoms. The pharmacophore models for both clusters are shown below (Figure 2).

### 2.4. Databases Search

Pharmacophore searches were conducted on the NCI compounds database and DrugBank FDA-approved drug database using each of the two pharmacophore models. Default settings were selected for Cluster A pharmacophore model. Partial matches of eight of 10 and nine of 10 were run on the NCI Database and DrugBank Database, respectively. This was due to a greater number of pharmacophore features elucidated in the Cluster B pharmacophore model than in the Cluster A pharmacophore model (ten, rather than seven), which increased the selectivity of the pharmacophore search. The partial matches, which included compounds with correspondence to at least eight of the 10 features for the NCI database search and nine of the 10 features for the DrugBank database search, allowed us to increase the number of hits generated.

#### 2.4.1. NCI Database Search

We searched an NCI compounds database containing 260,071 experimental compounds using the two pharmacophore models. When we searched the NCI compounds database with the five-of-five feature pharmacophore for Cluster A, 35 compounds were elucidated with 234 conformations. These 35 compounds contain all five pharmacophore features. When we searched the database with the eight-of-ten feature pharmacophore for Cluster B, 100 compounds were elucidated containing eight of the ten pharmacophore features, at minimum. Of the 135 total compounds found by the NCI database search, twelve top-scoring compounds were selected with greatest correspondence to the pharmacophore centers (Table 1).

#### 2.4.2. DrugBank Database Search

Similarly, we searched the DrugBank database containing 2356 FDA-approved drugs using the two pharmacophore models. The pharmacophore search on the DrugBank database using the five-of-five pharmacophore for Cluster A returned 57 drugs with 1974 conformations. These drugs share all five pharmacophore features. The pharmacophore search on the DrugBank database with the nine-of-ten pharmacophore for Cluster B elucidated 21 drugs containing nine of the ten pharmacophore features, at minimum.

### 2.5. Machine Learning Model

#### 2.5.1. Training Set Preparation

Three datasets were constructed to build and evaluate the classification model. The training set contained 43 PTCSCs and 42 non-PTCSCs. The training set contained the bulk of the known inhibitors so that the algorithm would have enough data to learn an accurate representation. Ten inhibitors were excluded from the training set, so that model could be evaluated on an independent set, one which did not influence the development of the model, to demonstrate that this model can be useful in identification of molecules which it has not seen before. The testing sets also included five times as many non-inhibitors as inhibitors. This was done to simulate the screen’s actual purpose; the elucidation of inhibitors in large molecular databases, where only one in many thousands to hundreds of thousands of molecules will end up having the desired activity. Another practice we wished to investigate is the control of drug screens for molecular weights. It is common practice to make sure the molecules in your inactive class, those which are not of interest, are of similar molecular weight to the molecules in your active class, those compounds which are of interest. The thought here being that molecular weight is a poor indicator of chemical activity and should not be used to bias model training. One could easily imagine a situation in which a drug screen has near perfect accuracy on a training or test set purely because the inactive molecules selected are of a wildly different size whether much smaller or larger. We aimed to investigate whether it is more useful to match the distribution of molecular weights to the active molecules in the training or testing set when designing a drug screen by including two test sets, one adapted for each situation. The molecular weight distribution in these sets are described in Table 2, Table 3 and Table 4 with Test A have a distribution representative of the active molecules in the training set and Test B, being representative of the distribution of active compounds in the test set.

#### 2.5.2. Model Development and Validation

Models were trained to classify between PTCSCs and non-PTCSCs. WEKA and TF were used for comparison of the machine learning platforms. Performance on the training set was evaluated on accuracy and AUC, although other metrics like false positive rate were also recorded and taken into consideration (Table 5). The models’ performance on outside data, that which had not been included in the training set, was evaluated largely on the false positive and false negative rates, due to the 5:1 sample bias against inhibitors molecules (Table 2). We felt this would indicate how the model would perform when used as a drug screen filtering through large datasets in which only a small number of molecules are of interest. The bias we artificially created will only be exaggerated by the application of our model to large molecular databases.

Table 5 and Table 6 list the training test results for the most powerful models we developed using WEKA and TF. The best performing model developed with WEKA achieved an accuracy of 85.88% and an AUC of 0.928 on the training set using an MLP architecture. The top performing model trained using TF, Model 4 (TF M4), achieved an accuracy of 94.12% with an AUC of 1.0.

Had TF M4 not been tested on a validation set we would think it to be our most powerful. However, it demonstrates poor accuracy on both our testing sets, 75.81% on Test A and 78.33% on Test B (Table 6). This is true for the WEKA MLP model as well, achieving only 75.00% accuracy on Test A and 70.00% accuracy on Test B, despite predicting with 85.88% accuracy on the training set. Both these models demonstrate over fitting: too strictly learning, adhering to, or memorizing, the training set at the cost of performance on other data sets, in this case Test A and Test B. Model 1 demonstrates the opposite problem, under fitting, where the model over generalizes the training set. Here, it creates a situation in which the model performs better on the testing sets than on the training set. Creating a successful machine learning model relies on balancing under and over fitting. A model that displays neither is no longer a model, it is a solution. Machine learning techniques are not meant to yield rigorous solutions, but rather approximations over the problem space presented in their training sets.

**Figure 3:**
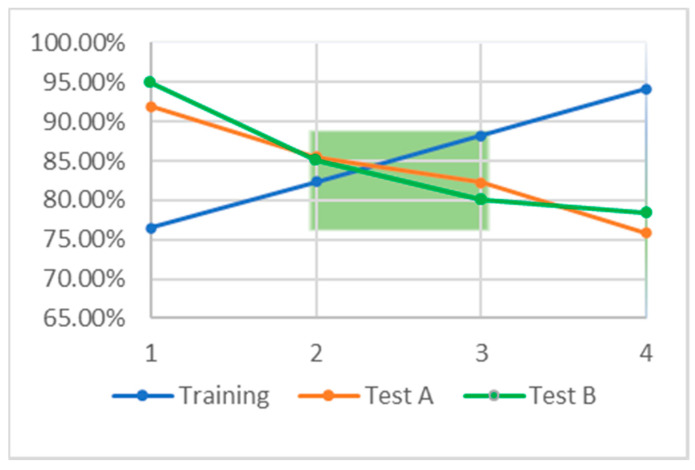
The relationship between accuracy on the training set, blue, and accuracy on the testing sets, orange and green. The green box indicates so called “goldilocks zone” in which the accuracy on the training and test sets is approximately equal. This region indicates the area over which there is minimal under and over training.

Models 2 and 3 are the most balanced, demonstrating accuracies in the mid 80% range for all three datasets. Figure 3 illustrates that these two balanced models fall between the other two developed in TF; they occupy the ML “goldilocks zone” (green box), illustrating the region in which under fitting and over fitting are minimized. Model 2 is slightly under fitting, because the testing set accuracy is slightly higher than that of the training set. Model 3 is the opposite, slightly over trained, as the training accuracy exceeds that of both testing sets.

Selecting which of these models to apply and how to apply them when searching a large molecular database is not straightforward. We describe two approaches, one either selects the single model they feel suits their problem or situation best, or uses multiple models in a committee approach, one in which multiple models are used as voters in a final decision. A single model should be selected if it greatly exceeds the performance of the other models developed in most or all the metrics being tracked. A committee approach often works very well if the models do not seem to obviously separate those themselves from each other, yet all perform well enough. We considered first the false negative rates on the test sets, and second the false positive rates on the test sets to determine which approach might be best in this situation. False negative rate was given priority because it demonstrates how often we will miss a molecule of the desired function, in this case how often a PTCSC will be classified as a PTCSC. This is the costliest form of error in this problem, because it describes a missed opportunity, a potential drug which will not be identified.

The false positive rate, while not as important as the false negative rate, is also of great importance, because it describes the frequency with which inactive compounds will end up being tested in vitro, or the rate at which molecules with no activity will be tested in vitro. The cost this adds to drug discovery can grow quickly when considering the quantity of false positives present when a screen is applied to a database with hundreds of thousands of molecules, a minute fraction of which will be active. 

Model 2 performed best when considering results across datasets, especially considering its correct identification of 9/10 inhibitors in the testing sets and relatively low false positive rate (Table 7).

The next step would be to use this trained model on a large molecular database, such as the set of all FDA approved drugs.

### 2.6. Model Deployment

After the development of both our pharmacophore and machine learning based QSAR models, we applied each of the three to the database of FDA approved drugs. Preprocessing of the FDA drug database for machine learning based predictions followed the same exact procedure as the other testing datasets, minus a label designating the molecules’ activity. This is the unknown, and the purpose of the models is to designate these labels, WEKA, or a corresponding probability of falling into the active class, TensorFlow. The pharmacophore modeling method yielded 95 molecules with possible activity. The output of the TensorFlow based deep learning model was a probability of activity on the target protein for each of the molecules. We included the 176 most probable molecules predicted by TF, all those which had a greater than 50% probability of activity according to the model. The WEKA model predicted 167 molecules in the set to be active on the target protein. The models predicted a combined total of 350 molecules to be active on our target. All three models agreed on 16 molecules (Table 8). Note that a majority of the selected compounds are antibiotics targeting the 30S ribosomal subunit of bacteria.

**Figure 4:**
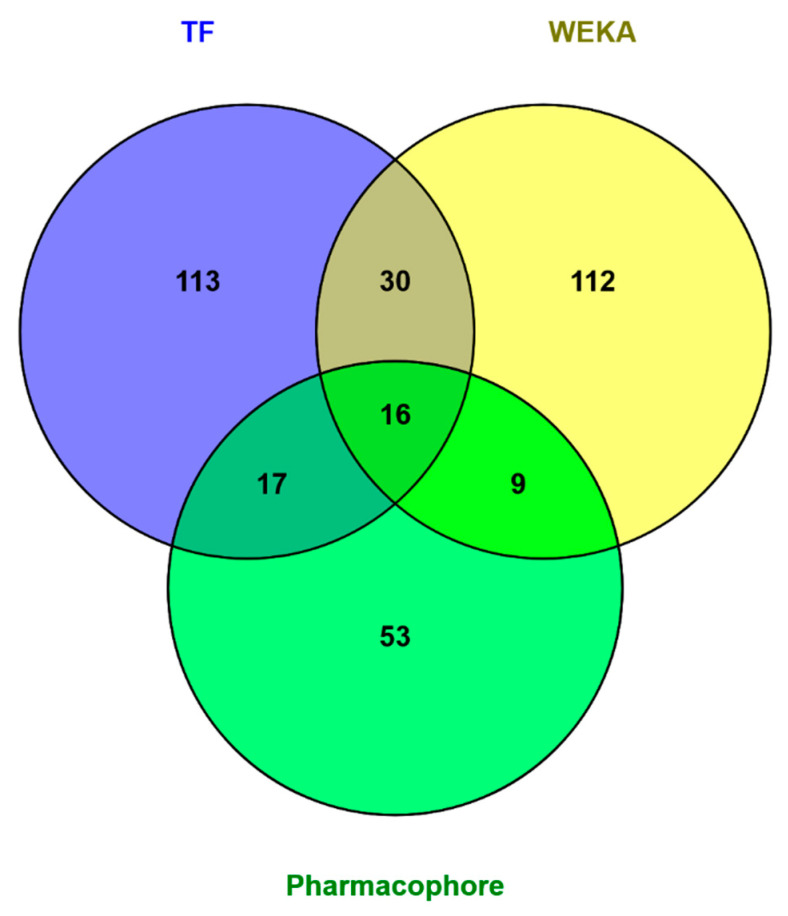
Overlap between molecules predicted to be active on the target proteins by different methods—pharmacophore-based and machine learning (ML) and deep learning (DL)-based. The three methods agree on 16 molecules. The largest overlap between two methods are the 46 molecules predicted to be active by both models developed in TensorFlow and WEKA.

The overlap between the TensorFlow model and the WEKA model predictions was most significant, sharing 46 of their combined 297 predictions (Figure 4). The next largest overlap between molecules predicted by two methods is between those chosen using pharmacophore modeling and TensorFlow, 33 molecules predicted to be active out of a combined 238 structures (Table 9). The overlap between the WEKA model and pharmacophore models is smallest, but still sizable at 25 molecules.

### 2.7. Building QSAR Models

**Figure 5:**
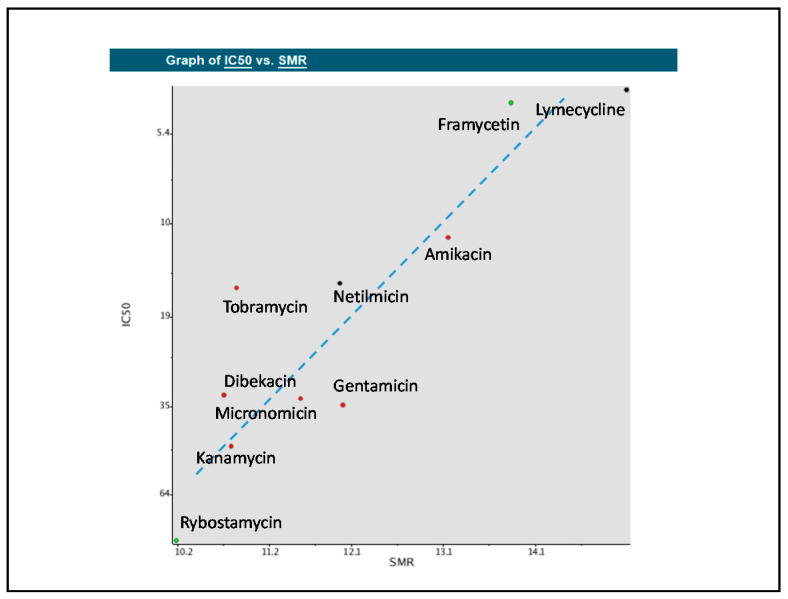
Quantitative structure-activity relationship (QSAR) model displaying the relationships between molecular refractivity (SMR) and the IC_50_ values of the compounds. A line of best fit was added to display the approximately linear relationship. The dots represent new compounds found in the consensus of pharmacophore search, WEKA machine learning, and TensorFlow deep learning prediction that these compounds would suppress premature termination codons (PTCs).

We developed QSAR models for 10 of the 16 consensus drugs selected by pharmacophore-based, WEKA-based and TensorFlow-based searches of the FDA-approved drug database that have the same target (30S ribosome) (see Supplementary Materials). We elucidated the QSAR descriptors most useful to our modeling problem to analyze their relation to inhibitory activity on the 30S ribosome. Molecular refractivity (including implicit hydrogens)—SMR—proved to be most suitable for this comparison, displaying the strongest linear relationship to IC_50_ for these compounds (Figure 5) [36].

**Figure 6:**
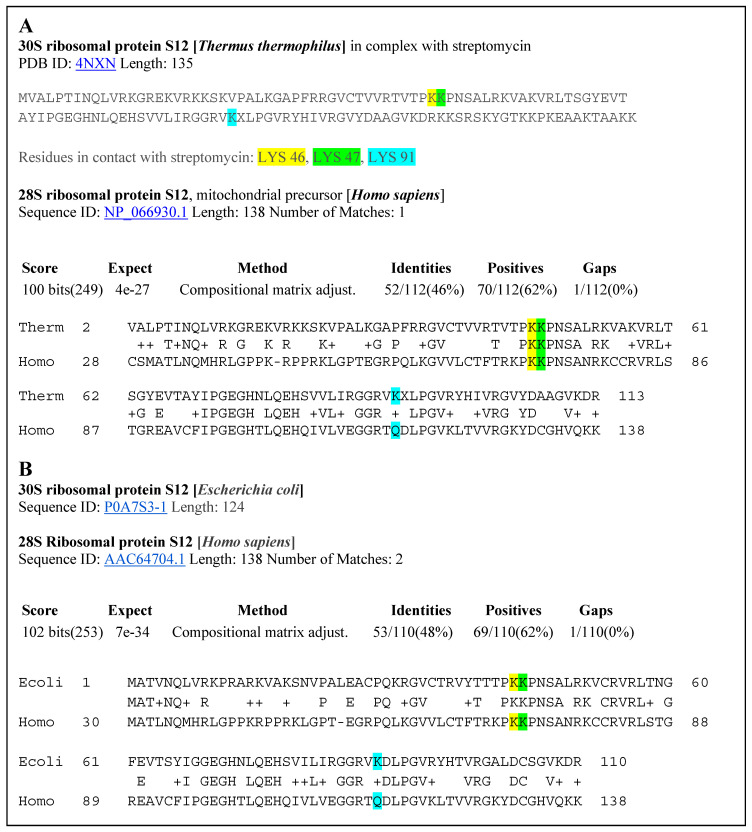
(**A**) Alignment of the 30S ribosomal protein S12 of *Thermus thermophilus* and the 28S ribosomal protein of *Homo sapiens*. (**B**) Alignment of the 30S ribosomal protein S12 of *Escherichia coli* with the 28S ribosomal protein S12 of *Homo sapiens*.

A reasonable question arose from the obtained results—how the inhibition of bacterial proteins in the 30S ribosomal subunit is related to inhibition of the analogous human proteins. We conducted a study of the possible homology of bacterial and human proteins and obtained this unexpected but very interesting result (Figure 6).

**Figure 7:**
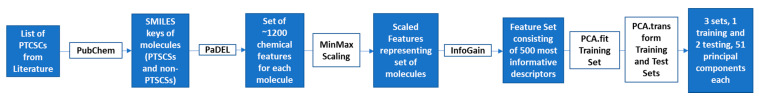
The preprocessing steps taken to prepare the datasets for use in machine learning.

The 30S ribosomal protein S12 sequence of *Thermus thermophilus* extracted from the crystal structure of this protein bound with the antibiotic streptomycin is shown at the top of Figure 7. The amino acids in contact with streptomycin are Lys46 (yellow), Lys47 (green), and Lys 91 (blue). The second sequence, below, is the analogous ribosomal sequence from *Escherichia coli*. BLAST searches of a possible alignment to human protein brought very interesting results. On a region spanning more than 110 amino acids in the 30S ribosomal protein S12 of *Thermus thermophilus*, there is a 46% identity alignment with the 28S ribosomal protein S12 of *Homo sapiens*. Alignment of the *Escherichia coli* and *Homo sapiens* ribosomal proteins produced a sequence identity of 48%. Such identities support the significant structural homology of these proteins in the region that is involved in binding of PTC-suppressing antibiotics. These results justify the use of IC_50_ values based on inhibition of the bacterial ribosomal protein for prediction of activity of PTC-suppressing drugs in human patients (Figure 6).

## 3. Discussion

Many compounds that have been developed to treat DMD by stimulating translational readthrough are limited, due to their low efficacy or high toxicity. Aminoglycosides, such as gentamicin and paromomycin, have been found to induce ototoxicity and nephrotoxicity when administered to patients [37]. The use of non-aminoglycosides to treat DMD is still being investigated; however, the efficacy of these compounds varies, due to the stop codon context and sequence specificity.

We implemented pharmacophore-based, ML, and DL approaches to address this issue, with the aim of identifying potent compounds, including FDA-approved compounds, with PTC-suppressing capability. A literature search was conducted to identify known PTC-suppressing compounds. We then clustered the chosen compounds by molecular fingerprints. Compounds within each cluster were structurally aligned. Using these alignments, we developed two pharmacophore models. These models were used to conduct a search on the NCI database and DrugBank database of FDA-approved drugs. We selected 16 FDA-approved drugs as a consensus of all three approaches. Similar studies may be done in the future to further identify PTC-suppressing compounds for the treatment of DMD using larger, more expansive databases, such as ChEMBL.

Based on our QSAR models, we observed approximately linear relationships between the biological activities and structural attributes of each of the compounds. Our QSAR models show that the inhibitory activity against the 30S ribosome of drugs found by the consensus of pharmacophore-based search, ML, and DL methods have a close to linear relationship with the descriptor of molecular refractivity. Moreover, we confirmed that the bacterial ribosomal proteins interacting with antibiotics have a significant homology with the human ribosomal proteins. This suggests an answer to the question of why these antibiotics cause PTC suppression in human patients.

We applied all three models to the database of FDA approved drugs and obtained interesting results. All three models, WEKA, TensorFlow, and Pharmacophore, agreed on 16 molecules. These FDA approved drugs are prime candidates for in vitro testing to start the process of repurposing them to treat DMD. Pharmacophore modeling is a better predictor of biological activity than are QSAR methods, because in QSAR, we are limited to compounds targeting the same molecule. The molecules contained in the overlap between the pharmacophore model and TensorFlow model predictions sets are slightly more promising than the WEKA ML models because the TensorFlow DL model performed better than the WEKA model on independent testing sets.

Molecules predicted to have activity by more than one method should, in general, be weighted significantly higher than those which are only labeled active by only one model. Ensemble modeling methods, those in which multiple models are developed to make a single prediction, have been demonstrated to be more powerful than prediction methods dependent on only one model. Using an ensemble modeling method to make predictions is analogous to getting multiple professional opinions on a subject matter before making a decision. It allows for the prediction to account for many, well informed opinions, selecting the options which make sense from multiple perspectives.

Our analysis presents several potent compounds with PTC-suppressing ability. Ten of the 16 compounds identified using pharmacophore, ML, and DL models target the 30S ribosomal subunit, the primary target of existing PTC-suppressing compounds, indicating that they are viable options for repurposing for DMD treatment. Of the other six compounds, diazolidinyl urea, steviolbioside, streptozocin, and rutin have similar mechanisms of action (inhibition of DNA/protein synthesis), and their targets have similar structures to the 30S ribosomal subunit. This indicates that these compounds may have potential PTC-suppressing ability as well. With regard to the structures of the compounds found from all three approaches, 10 of the 16 compounds contain a 2-deoxystreptamine (2 DOS), which has been identified as a key structural feature in novel aminoglycoside structures [8]. In general, the structures of the compounds indicate PTC-suppressing ability. These compounds warrant further analysis of their pharmacokinetic properties and experimental validation of the development of these compounds for drug design.

## 4. Materials and Methods

### 4.1. Building of a Database

A series of PTC-suppressing compounds previously investigated for the treatment of DMD were obtained from the public sources [8,9,10,11,12,13,14,15,16,17,18,19,20,21]. A total of 37 compounds were selected, including aminoglycosides and oxadiazoles.

### 4.2. Similarity Clustering

MOE (Molecular Operating Environment, Chemical Computing Group, Montreal, Canada) was used to perform similarity clustering. The main objective of similarity clustering is to separate compounds into subsets by their molecular fingerprints, based on the hypothesis that compounds with structural similarity will have similar binding properties. Examples of common molecular fingerprints include MACCS (Molecular ACCess System) and GpiDAPH3 (graph of pi-system-donor-acceptor-polar-hydrophobe three-point pharmacophore). The MACCS fingerprint encodes molecular structures in a bit string representing the presence or absence of sub-structural features [38]. GpiDAPH3 is calculated from the 2D molecular graph of a three-point pharmacophore [38]. The following three atomic properties: “is a hydrophobic”, “is a donor”, “is an acceptor” are computed to assign each atom to one of eight atom types [38]. Previous studies have ranked the GpiDAPH3 fingerprint above the MACCS fingerprint in terms of performance on datasets [38]. For the similarity clustering in this study, the GpiDAPH3 fingerprint was used. The SO (similarity and overlap) value selected was 0.45.

### 4.3. Flexible Alignment

The primary objective of flexible alignment is to identify the overlap of molecular features in selected compounds. Prior to pharmacophore elucidation, flexible alignment is a necessary step to superimpose the structures of selected compounds. Using the Flexible Alignment module from the Compute application in MOE, we performed flexible alignment separately for each cluster, given that each cluster was composed of distinct compounds with distinct chemical structures. The following parameters were used: Iteration Limit = 200, Failure Limit = 20, Energy Cutoff = 10.

### 4.4. Development of a Pharmacophore Model

The Query Editor of the Pharmacophore module of the Compute application in MOE was employed to build pharmacophore models for both clusters’ alignments. The consensus pharmacophore models allowed us to select possible pharmacophore centers. The following pharmacophore centers were applied: H-bond donors (Don), H-bond acceptors (Acc), H-bond donors/acceptors (Don & Acc), and hydrophobic features (Hyd). The following parameters were used: Tolerance = 1.2, Threshold = 50%, and Consensus Score = Weighted Conformations. For the completion of the pharmacophore model, we used centers that belong to 100% of the superimposed compounds for Cluster A and 80% of the superimposed compounds for Cluster B. Based on the generated models, specific features were added accordingly to areas with high density of nitrogen atoms (Don), areas with high density of oxygen atoms (Acc), areas with high density of nitrogen and oxygen atoms (Don & Acc), or areas with high density of hydrophobic atoms (Hyd).

### 4.5. Databases Search

The Search window of the Pharmacophore module of the Compute application in MOE was employed to conduct searches of two databases: the NCI open database and the DrugBank database.

#### 4.5.1. NCI Database Search 

We prepared a conformational database from the NCI open database containing 260,071 experimental compounds, using the Conformational Search module of the Compute application in MOE. A pharmacophore search was then conducted on this database using the pharmacophore queries generated from the previous step. The default settings were used for Cluster A. For Cluster B, a partial match of 8 of 10 was run to increase the number of hits generated.

#### 4.5.2. Drugbank Database Search 

We also created a conformational database from the DrugBank database containing all 2356 FDA-approved drugs using the Conformational Search module of the Compute application in MOE. A pharmacophore search was conducted on this database, in the same way as with the NCI open database. The default settings were used for Cluster A. For Cluster B, a partial match of 9 of 10 was run to increase the number of hits generated.

Top-scoring compounds were identified based on the following criteria: a) the compounds must be within the ligand shape volume so that no atom centers lie beyond this volume; b) the highest-scoring compounds should have the greatest correspondence to the geometry of the selected pharmacophore centers.

### 4.6. Machine Learning Model

#### 4.6.1. Training and Test Set Preparation

Three data sets where constructed. One was for training the model, containing 43 PTCSCs and 42 random molecules with no documented affinity for the protein. The 37 PTCSCs used in pharmacophore-based methods and an additional 6 PTCSCs found from literature—lividomycin, neomycin sulfate, spectinomycin, isepamicin, clarithromycin, and telithromycin—were selected as inhibitors [39,40,41,42,43]. Non-inhibitors were selected to match the distribution of molecular weights observed in the inhibitor class in order to ensure that molecular weight—a characteristic we believe would bias the algorithm in a non-functionally impactful way—does not influence the model’s training parameters (Table 2).

The other two sets contained a common set of 10 inhibitors gathered from literature, none of which appeared in the training set: viomycin, roxithromycin, troleandomycin, tigecycline, omadacycline, demeclocycline, linezolid, eravacycline, chloramphenicol, and capreomycin [42,44,45,46,47]. This was necessary to ensure that the sets contained enough distinct compounds that could be used as independent testing sets for model validation. The two testing sets differ in their distributions of molecular weights with respect to those of the non-PTCSCs they contain. The first set contained a set of non-inhibitors whose distribution of molecular weights mimics the training set, Set A (Table 3), while the second set of non-inhibitor molecular weights matches the testing set, Set B (Table 4). Set B was designed to have approximately 5 non-inhibitors for every one of the 10 known inhibitors in the testing set.

SMILES (simplified molecular-input line-entry system) representations of all molecules were collected from PubChem and used to compute QSAR molecular descriptors and fingerprints using the PaDEL online descriptor calculator [48]. Descriptors for the training set were uploaded to WEKA (Waikato Environment for Knowledge Analysis, University of Waikato, New Zealand) [49] and ranked by Information Gain (IG) with respect to the label of active/inactive. The 500 most informative columns were kept and ranked. The same 500 descriptors were isolated for the two testing sets. All three sets were MinMax scaled (Equation (1)), using the minimum and maximum values of each column presented in the training set.

X_sc_ = (X − X_min_)/(X_max_ − X_min_) (1)

Equation (1). The formula for MinMax scaling where X_sc_ is the scaled value, X is the actual value for a feature in the column before scaling, X_min_ is the minimum value for that feature in the training set, and X_max_ is the maximum value for that feature in the training set.

The scaled sets were then reduced to their principal components using Numerical Python--a library consisting of multidimensional array objects and a collection of routines for processing those arrays (NumPy). Formulation of the principal components was performed using only the training set, with the formula derived from the training set applied to the training set and each of the testing sets. Principal component analysis (PCA) is a mathematical technique by which the unique basis sets of a set of numbers can be determined. It is commonly used to reduce the noise in a data set, allowing machine learning algorithms to converge at a minimal error more quickly. The resulting data sets contained 51 principal components representing each molecule (Figure 7). A label designating activity or lack thereof was added manually.

#### 4.6.2. Model Development

Machine learning was performed using both WEKA [49] and TensorFlow (TF) [50], comparing results obtained using each program. WEKA is a popular GUI typically used to accomplish basic ML tasks [49]. It is also equipped with number of preprocessing and feature selection functions, such as the previously mentioned IG and PCA [49]. TF is a more sophisticated ML platform requiring knowledge of a computer language, Python in this case, which allows for greater customization of the algorithms [50]. TF was used with Keras backend for access to its neural network libraries [50]. Models were trained through 10-fold cross validation and evaluated on their accuracy and area under receiver operating characteristic curve (AUC), with respect to the training set and the false negative and false positive rates on the testing sets. Modeling in WEKA was performed using default settings for all architectures attempted with multilayer perceptron (MLP) performing best across the sets. Modeling in TF was attempted with neural networks containing between 2 and 5 layers, with the best results achieved using a 3-layer neural network with parameters illustrated in Table 10.

### 4.7. Building QSAR Models

We prepared a database containing the names and experimental IC_50_ values for 10 of the 16 drugs identified as a consensus of the pharmacophore-based, ML, and DL search of the DrugBank database. The IC_50_ values were obtained from literature [28,29,30,31,32,33,34,35]. This database was submitted to the Structure-Activity Report (SAReport) editor of the QuaSAR module of the Compute Application in MOE in order to develop QSAR (Quantitative structure-activity relationship) models. The primary objective of developing QSAR models was to compare the therapeutic potential of the compounds found by the pharmacophore search to those currently used to treat DMD. QSAR was employed to identify the relationship between selected descriptors, which quantify the physicochemical properties of the compounds, and the IC_50_ values of the compounds, which describe the biological activity of the compounds. Descriptors displaying the strongest linear relationship with the IC_50_ values of the compounds were included in the QSAR models.

## Figures and Tables

**Table 1:** Representatives of the compounds selected using the NCI compounds database.

#	Compound Name	Chemical Formula	NSC ID	2D Structure	Cluster
1	2-[Diethyl-[(4-hydroxy-6-oxo-1H-pyrimidin2-yl)sulfanyl]stannyl] sulfanyl-4-hydroxy-1H-pyrimidin-6-one	C_12_H_16_N_4_O_4_S_2_Sn	NSC 356199	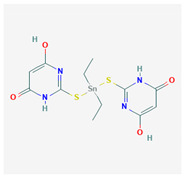	A
2	5-[(E)-Hydroxyimino methyl]-2-methoxy-3-methyl-6-[[3,4,5-trihydroxy-6-(hydroxymethyl)oxan-2-yl]amino]pyrimidin-4-one	C_13_H_20_N_4_O_8_	NSC 607158	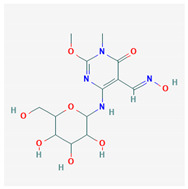	A
3	2-[2-Hydroxyethyl (methyl)amino]-1-[3,4,5-trihydroxy-6-(hydroxymethyl) oxan-2-yl]-5,6,7,8-tetrahydroquinazolin-4-one	C_17_H_27_N_3_O_7_	NSC 652577	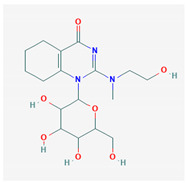	A
4	2-[[4-Amino-1-[3,4-dihydroxy-5-(hydro xymethyl)oxolan-2-yl]pyrimidin-2-ylidene]amino]-3-hydroxypropanoic acid	C_12_H_18_N_4_O_7_	NSC 164854	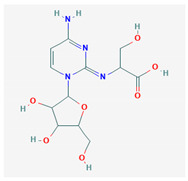	B
5	Barbaloin	C_21_H_22_O_9_	NSC 227189	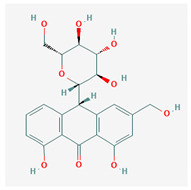	B
7	Salicin	C_13_H_18_O_7_	NSC 5751	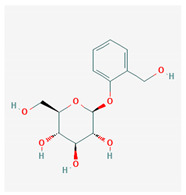	B
8	Shikonin glucoside	C_22_H_26_O_10_	NSC 289509	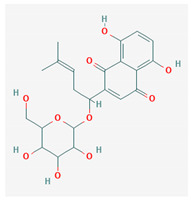	B
9	Hyperoside	C_21_H_20_O_12_	NSC 407304	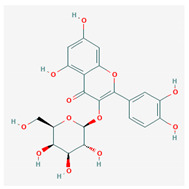	B
10	2-(Hydroxymethyl)-6-[5-(hydroxymethyl)-4-(methoxymethyl)-2-methylpyridin-3-yl]oxyoxane-3,4,5-triol	C_15_H_23_NO_8_	NSC 638029	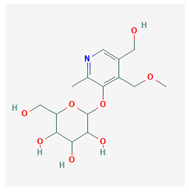	B
11	Aloin A	C_21_H_22_O_9_	NSC 374116	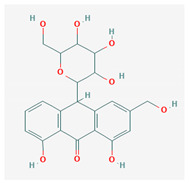	A, B
12	1,3,8-Trihydroxy-6-(hydroxymethyl)-10-[3, 4,5-trihydroxy-6-(hydroxymethyl)oxan-2-yl]-10H-anthracen-9-one	C_21_H_22_O_10_	NSC 658575	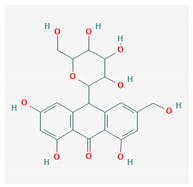	A, B

**Table 2:** The molecular weight distribution in the training set. The inactive set, inactive meaning non-inhibitor, contains one less molecule in the 400–500 g/mol range, because said molecule proved to be problematic later in preprocessing.

Molecular Weight Range g/(mol^−1^)	Active Training	Inactive Training
200–300	5	5
300–400	4	4
400–500	11	10
500–600	11	11
600–700	3	3
700–800	4	4
800–900	3	3
900–1000	1	1
1000–1200	1	1
**Total**	43	42

**Table 3:** The number of molecules in each molecular weight range in set Test A, the testing set whose non-inhibitor molecular weight range distribution as described in the table reflects those in the training set. The number of molecules needed for each range was multiplied by 50/43 and rounded to the nearest whole number in order to reach the desired 50 non-inhibitor molecules in this set, while keeping the same distribution of molecular weights as the training set.

Molecular Weight Range/g(mol^−1^)	Active Training	Before Rounding	Inactive Test A
200–300	5	5.96	6
300–400	4	4.76	5
400–500	11	13.1	13
500–600	11	13.1	13
600–700	3	3.57	4
700–800	4	4.76	5
800–900	3	3.57	4
900–1000	1	1.19	1
1000–1200	1	1.19	1

**Table 4:** The testing set, which is composed of molecules whose distribution of molecular weights in the non-inhibitor class matches that of the testing set. This means that there is a direct relationship between the number of inhibitor molecules in each molecular weight range as described in the table and the number of non-inhibitors in that molecular weight range. In this case there are 5 non-inhibitors for every inhibitor in the range.

Molecular Weight Range/g(mol^−1^)	Active Testing	Inactive Test B
200–300	0	0
300–400	3	15
400–500	1	5
500–600	4	20
600–700	1	5
700–800	0	0
800–900	1	5
900–1000	0	0
1000–1200	0	0

**Table 5:** Results for model training.

Model	Accuracy	AUC	Precision	Recall	False Negatives	False Positives
WEKA MLP	85.88%	0.928	0.878	0.837	7	5
TF M1	76.47%	0.7929	0.6667	0.8571	1	3
TF M2	82.35%	0.9357	1	0.7	3	0
TF M3	88.24%	0.9857	0.7778	1	0	2
TF M4	94.12%	1	0.875	1	0	1

**Table 6:** Results for model training with the Tests A and B.

Model	Training	Test A	Test B
TF M1	76.47%	91.94%	95.00%
TF M2	82.35%	85.46%	85.00%
TF M3	88.24%	82.26%	80.00%
TF M4	94.12%	75.81%	78.33%
WEKA MLP	85.88%	75.00%	70.00%

**Table 7:** Listing the false positive and false negative rates with respect to the inhibitor class of the testing sets.

Model	False Negative Rate	False Positive Rate
2 Test A	0.1	0.15
2 Test B	0.1	0.16
4 Test B	0.2	0.22
4 Test A	0.2	0.25
1 Test B	0.3	0
1 Test A	0.3	0.038
3 Test A	0.3	0.098
3 Test B	0.3	0.18
WEKA Best MLP Test A	0.3	0.19
WEKA Best MLP Test B	0.3	0.24

**Table 8:** Molecules predicted active by all three models: pharmacophore, TensorFlow, and Waikato Environment for Knowledge Analysis (WEKA). IC_50_ experimental values were gathered from the literature [28,29,30,31,32,33,34,35].

Drug Name	2D Structure	Drug Class and Activity	IC_50_ (μM)	Drug Target
Amikacin	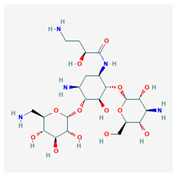	Aminoglycoside antibiotic	11	30S ribosomal subunit
Diazolidinyl urea	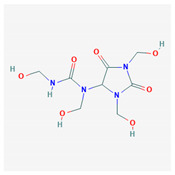	Antimicrobial preservative and that acts as an antibacterial agent	55	DNA
Dibekacin	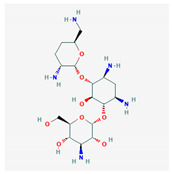	Aminoglycoside antibiotic	32.3	30S ribosomal subunit
Framycetin	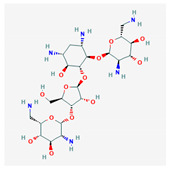	Aminoglycoside antibiotic	4.38	30S ribosomal subunit
Gentamicin	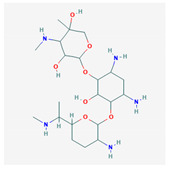	Aminoglycoside antibiotic	34.6	30S ribosomal subunit
Kanamycin	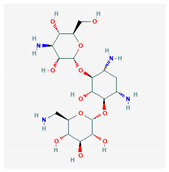	Aminoglycoside antibiotic	46	30S ribosomal subunit
Lactulose	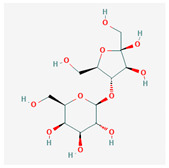	Synthetic disaccharide derivative of lactose	10	Evolved beta-galactosidase subunit alpha in Escherichia coli strain K12
Lymecycline	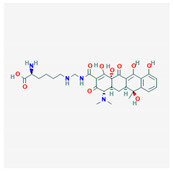	Tetracycline broad-spectrum antibiotic	4	30S ribosomal subunit
Micronomicin	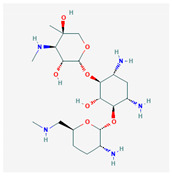	Aminoglycoside antibiotic	33	30S ribosomal subunit
Netilmicin	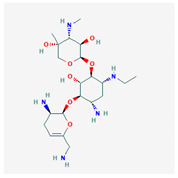	Aminoglycoside antibiotic derived from sisomicin	15	30S ribosomal subunit
Regadenoson	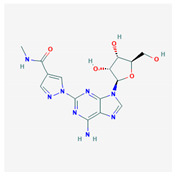	Adenosine receptor agonist	1.2	A2A adenosine receptor
Ribostamycin	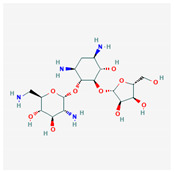	Aminoglycoside antibiotic	87.5	30S ribosomal subunit
Rutin	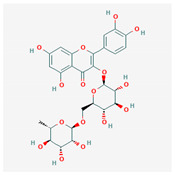	Flavonoid that exhibits antibacterial, anti-oxidant, anti-tumor, and anti-inflammatory activity	3.53	DNA topoisomerase IV
Steviolbioside	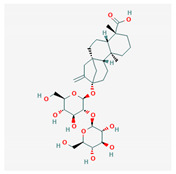	Beta-d-glucoside with moderate antituberculosis activity against the M. tuberculosis strain H37RV	48.2	M. tuberculosis strain H37RV
Streptozocin	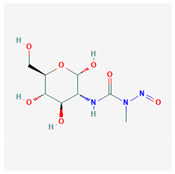	Antibiotic used as an antineoplastic agent to treat metastatic pancreatic islet cell carcinoma	11.7	Cytosine moieties of bacterial DNA
Tobramycin	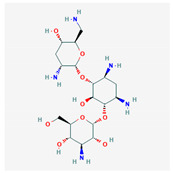	Aminoglycoside antibiotic	15.5	30S ribosomal subunit

**Table 9:** Drugs predicted active by pharmacophore search and TensorFlow.

Common Drugs in TF and Pharmacophore-Search		
Sorbitol	Demeclocycline	Lactulose
Zanamivir	Nelarabine	Dibekacin
Benserazide	Sarecycline	Rutin
Doxycycline	Riboflavin	Steviolbioside
L-Cysteine	Lincomycin	Micronomicin
Methacycline	Chlortetracycline	Gentamicin
Esculin	Kanamycin	Netilmicin
Spectinomycin	Framycetin	Streptozocin
Clofarabine	Ribostamycin	Diazolidinylurea
Cefotetan	Amikacin	Lymecycline
Tetracycline	Tobramycin	Regadenoson

**Table 10:** Illustrating training parameters which led to the best results across all three datasets using TF. The layer densities and activation functions are listed in the order they appear; the layer of density 50 relies on a sigmoid activation function, density 25 corresponds to the elu activation function, and 1 to another sigmoidal layer.

Layer Density	Layer Activation	Epochs	Optimizer
50, 25, 1	sigmoid, elu, sigmoid	50	adagrad

## Supplementary Materials

The following are available online, Table S1: Drugs selected by Pharmacophore-based, ML-based and DL-based search in the FDA-approved drugs database.
