# Supplementary material for: Prediction of Premature Termination Codon Suppressing Compounds for Treatment of Duchenne Muscular Dystrophy Using Machine Learning
Source: Molecules. 2020 Aug 26;25(17):3886. doi: 10.3390/molecules25173886 (PMC7503396; doi:10.3390/molecules25173886)
Supplement: Supplementary file 1 [file molecules-25-03886-s001.pdf]

Prediction of Premature Termination Codon Suppressing Compounds for Treatment of Duchenne Muscular Dystrophy using Machine Learning

Kate Wang et al.

Supplemental Table S1. Drugs selected by Pharmacophore-based, ML-based and DL-based search in the FDA-approved drugs database

Pharmacophore	WEKA	TF
1-Palmitoyl-2-oleoyl-sn-glycero-3-(phospho-rac-(1-glycerol))	5-O-phosphono-alpha-D-ribofuranosyl diphosphate	Acarbose
Amikacin	Acetylcarnitine	Acetarsol
Arbutamine	Acetylcholine	Adenosine
Benserazide	Acyclovir	Aldehydo-N-Acetyl-D-Glucosamine
Bisoprolol	Adefovir dipivoxil	Alendronic acid
Brivudine	Alfentanil	Alginic acid
Cefamandole	Alitretinoin	alpha-Arbutin
Cefdinir	Azithromycin	Amikacin
Cefixime	Balsalazide	Amiloride
Cefonicid	Bethanechol	Arbutin
Ceforanide	Bicalutamide	Ascorbic acid calcium salt
Cefotetan	Calcium glubionate	Auranofin
Ceftibuten	Cangrelor	Azacitidine
Ceftolozane	Capecitabine	Benserazide
Cerivastatin	Carbamoylcholine	Besifloxacin
Chlortetracycline	Carisoprodol	beta-L-fructofuranose
Cilastatin	Chlorobutanol	Bictegravir
Citicoline	Cidofovir	Bismuth subgallate
Cladribine	Clodronic acid	Bleomycin
Clarithromycin	Colistimethate	Bortezomib
Clindamycin	Cyclandelate	Bromotheophylline
Clofarabine	Dexpanthenol	Calcium threonate
Cromoglicic acid	Edoxudine	Capecitabine
Demeclocycline	Elbasvir	Capreomycin
Diaminopropanol tetraacetic acid	Erdosteine	Carbidopa
Diazolidinylurea	Ethchlorvynol	Carbocisteine
Dibekacin	Ethinamate	Carboplatin
Dinoprostone	Famotidine	Cefotetan

Dipyridamole
Doripenem
Doxycycline
Eliglustat
Eluxadoline
Empagliflozin
Eribulin
Ertapenem
Erythromycin
Esculin
Fenoterol
Flavin mononucleotide
Framycetin
Gadobenate
Gadopentetic acid
Gentamicin
Gluconic acid
Ibandronate
Kanamycin
Lactulose
Latamoxef
L-Cysteine
Lincomycin
Lisinopril
Lymecycline
Mangafodipir
Mannitol busulfan
Menadiol diphosphate
Methacycline
Micronomicin
Mitoxantrone
Moxifloxacin
Nadolol
Nelarabine
Netilmicin
Pantethine
Pemetrexed
Pentostatin
Peramivir
Plerixafor
Pravastatin
Protokylol

Fidaxomicin
Flavin adenine dinucleotide
Flurandrenolide
Fluticasone
Fondaparinux
Fosaprepitant
Fostamatinib
Gabapentin enacarbil
Gemifloxacin
Glecaprevir
Gluconic Acid
Glycine betaine
Hexetidine
Hyaluronic acid
Hydroxyethyl cellulose
Idoxuridine
Imidurea
Inulin
Isavuconazole
Isotretinoin
Lamivudine
Leflunomide
Levocarnitine
Linagliptin
Lynestrenol
Medronic acid
Memantine
Methacholine
Methenamine
Methohexital
Methylchloroisothiazolinone
Methylprednisone
Mupirocin
Nizatidine
Norgestrel
Novobiocin
Nystatin
Opicapone
Oritavancin
Pamidronic acid
Pantothenic acid
Pargyline

Chlormerodrin
Chlortetracycline
cis-Platin
Clofarabine
Crisaborole
Cytarabine
Dacarbazine
Danthron
Decitabine
Delafloxacin
Demeclocycline
Desflurane
Deslanoside
Dexpanthenol
Dextrothyroxine
D-glucose
Diazolidinylurea
Dibekacin
Diflorasone
Diflunisal
Dihydralazine
Diosmin
Dipentamethylenethiuram disulfide
Disulfiram
Ditiocarb Zinc
Dopamine
Doxorubicin
Doxycycline
Droxidopa
Edetic Acid
Eflornithine
Emtricitabine
Epirubicin
Eravacycline
Esculin
Ferrostrene
Floxuridine
Fludarabine
Fludeoxyglucose
Flumethasone
Framycetin
Gadobutrol

Regadenoson
Riboflavin
Ribostamycin
Rifabutin
Rolitetracycline
Rosuvastatin
Rutin
Sarecycline
Somatostatin
Sorbitol
Spectinomycin
Steviolbioside
Streptozocin
Sulfoxone
Temocillin
Tetracycline
Tezacaftor
Tobramycin
Travoprost
Unoprostone
Valganciclovir
Valrubicin
Vilanterol
Zanamivir

Pentafluoropropane
Pentobarbital
Pentosan Polysulfate
Permethrin
Pibrentasvir
Plicamycin
Polythiazide
Pralatrexate
Pyrophosphoric acid
Ranitidine
Rivaroxaban
Roxithromycin
Saxagliptin
Selegiline
Semaglutide
Sevoflurane
Sitagliptin
Sofosbuvir
Stavudine
Stiripentol
Sugammadex
Sulfasalazine
Sultamicillin
Tacrolimus
Taurolidine
Telavancin
Telbivudine
Terbinafine
Tesamorelin
Thiopental
Ticagrelor
Tioconazole
Tretinoin
Triclofos
Troleandomycin
Trypan blue free acid
Valaciclovir
Vancomycin
Vildagliptin
Vitamin A
Voriconazole
Xanthinol
Zalcitabine

Gadodiamide
Galactose
Gaxilose
Gemcitabine
Gemifloxacin
Gentamicin
Gluconolactone
Glucosamine
Glycol salicylate
Hesperidin
Hyaluronan
Hydrogen peroxide
Imidazolidinyl Urea
Imipenem
Inositol
Iohexol
Iomeprol
Iopamidol
Iotalamic Acid
Ioversol
Ioxitalamic acid
Isoprinosine
Isosorbide mononitrate
Ixazomib
Kanamycin
Lactose
Lactulose
L-Ascorbic Acid
L-Cysteine
Levodopa
Levonordefrin
Levothyroxine
Lincomycin
Liothyronine
Liotrix
L-Lysine
Lodoxamide
Lymecycline
Magnesium
Magnesium ascorbate
Mannitol
Methacycline
Methyldopa

Zidovudine
Paromomycin
Lactose
Sucrose
Gaxilose
Gemcitabine
Fludarabine
Delafloxacin
Eflornithine
Azacitidine
Cytarabine
Streptomycin
Alginic acid
Emtricitabine
Trifluridine
Plazomicin
Bleomycin
Hesperidin
Diosmin
Floxuridine
Epirubicin
Doxorubicin
Gadobutrol
Bictegravir
Amiloride
Temozolomide
Decitabine
Deslanoside
Teriflunomide
Cilastatin
Citicoline
Cladribine
Clindamycin
Erythromycin
Ibandronate
Temocillin
Tezacaftor
Valganciclovir
Amikacin
Diazolidinylurea
Dibekacin
Framycetin
Gentamicin

Micronomicin
Miglitol
Miglustat
Minocycline
Mithramycin
Nedaplatin
Nelarabine
Netilmicin
Niacinamide ascorbate
Nitroferricyanide
Norepinephrine
Omadacycline
Ouabain
Oxaliplatin
Oxidronic acid
Oxytetracycline
Paromomycin
Perboric acid
Perflubutane
Perflutren
Plazomicin
Plecanatide
Prussian Blue
Ramidronic Acid
Regadenoson
Ribavirin
Riboflavin
Ribostamycin
Risedronic acid
Rutin
Sapropterin
Sarecycline
Selenomethionine
Sodium ascorbate
Sodium aurothiomalate
Sorbitol
Sparfloxacin
Spectinomycin
Steviolbioside
Streptomycin
Streptozocin
Sucralfat
Sucrose

Kanamycin	Tavaborole
Lactulose	Tegafur
Lymecycline	Tegafur-uracil
Micronomicin	Temozolomide
Netilmicin	Teriflunomide
Regadenoson	Tetracycline
Ribostamycin	Thimerosal
Rutin	Thiram
Steviolbioside	Titanium dioxide
Streptozocin	Tobramycin
Tobramycin	Triamcinolone
	Trifluridine
	Triflusal
	Trovafloxacin
	Vaborbactam
	Vidarabine
	Viomycin
	Xylose
	Zanamivir
	Zoledronic Acid
